# Nanoengineering and Surface Modifications of Dental Implants

**DOI:** 10.7759/cureus.51526

**Published:** 2024-01-02

**Authors:** Ahmed Alamoudi

**Affiliations:** 1 Oral Biology, King Abdulaziz University, Jeddah, SAU

**Keywords:** implantology, implant surface, implant surface modification, implant nanoengineering, dental implant

## Abstract

Dental implants are one of the most important and successful advancements in modern dentistry. One aspect of dental implant design that influences the rate and degree of osseointegration is implant surface features. Nano-engineering techniques are anticipated to improve titanium dentistry implants' surface characteristics, which in turn promote peri-implant osteogenesis. In this paper, we review the recent advances in nanosurface engineering techniques for enhancing the bioactivity of dental implants.

## Introduction and background

With more people becoming familiar with the advantages of implant-supported prostheses, dental implants are experiencing a considerable rise nowadays [1]. Dental implants are quickly changing from an optional dental procedure to an increasingly accepted and in-demand method of treatment for tooth loss due to their durability and scientific advancement [1]. The cost of dental implants can vary widely depending on several factors, including implant location, the number of implants needed, and the type of implant used [1]. However, it is important to keep in mind that dental implants are a long-term solution that can provide many benefits such as esthetics, function, and patient comfort and acceptance compared to other tooth replacement options [1, 2]. Commercially pure (cp) titanium (1-4 grades) or alloys of titanium like Ti-6Al-4V make up most dental implants nowadays [1, 2]. The patient's health status, dental and surgical technique, implant material, implant design, and implant surface all affect an implant's success [1, 2]. One aspect of dental implant design that influences the rate and degree of osseointegration is implant surface features [1, 2]. The structure of the implant surface itself affects the extent to which the implant surface physically aligns with the bone, how fast this bone accretion develops, and the biomechanical form of the bone/implant relationship [1, 2]. A variety of surface topographies at the micron scale promote extracellular matrix production and mineralization as well as adherent osteoblast development [3]. It is demonstrated that the nano-surface design of dental implants significantly increases adherent cells' production of extracellular matrix and offers faster osseointegration compared to the machined surface of the implant [3].

Although micro-roughness is thought to be the best way for creating a proper interface between an implant and the surrounding bone, nano-engineering is becoming a brand-new base for progressively enhancing the bioactivity of dental implants [4]. Numerous investigations have demonstrated that the nano-scale surface morphology of Ti implants gives increased bioactivity and outperforms the micro-roughness, both in vitro and in vivo contexts [4]. Several techniques, including micro-machining, plasma treatment, electrochemical anodization, particle blasting, polishing/grinding, and chemical etching have been used to create nano-engineered Ti dental implant surfaces [5]. 

## Review

Nanotechnology

The technique and research associated with the concept, production, description, and use of tools and substances whose tiniest structural and fundamental arrangement, in minimum, a dimension, is on the nanoscale scale (one billionth of a meter) can be referred to as nanotechnology [6]. As a result, substances used in nanotechnology have nanoscale topographies or are made of elements with a nanoscale size ranging from 1 to 100 nm (10-9 m) [7]. An analysis of the nanoscale characteristics of the adjacent bone by nano-indentation showed that the cell condition was greatly improved around the hydroxyapatite (HA)-coated implants [8]. Nanotechnology can use one-dimensional notions like nanodots and nanowires or it can self-assemble more complicated forms such as nanotubes. Substances can also be categorized as nanocrystals, nanoparticles, nanostructures, nanolayers, and nanofibers based on their composition and shape [7]. 

The range of biomechanical functionality in living organisms ranges from the observable to the atomic or nanoscale [9]. The scale of nanotechnology, which runs from 1-100 nm, has attracted much interest in both research and general media [9]. The nanotopography of implants is believed to affect cell-implant contacts at the cellular and structural levels. Whereas the microtopography of the implant surface has been postulated to impact the microscopic scale of osseointegration [9, 10]. Biomedical engineers began concentrating on dental implant designing at the nanoscale just recently [11]. Manufacturers have found that some parts of their implants' topography are nanoscale [12, 13]. In addition to modifications in the surface texture, modifications in the surface's chemical composition also contribute significantly to a rise in the surface's energy [11]. Consequently, modifications in nanotopography have an impact on the matter of the structure, composition, and physiology, increasing the adherence of osteogenic cells and possibly facilitating osseointegration [14]. The variable osteoconductivity of implant materials at the micro and nanoscale has been postulated to affect osteoblast function. Ongoing improvements in restorative dentistry surface morphology are essential to enhance results in complex medical circumstances, such as early loading protocols, prompt implantation following a tooth extraction, and patients having weak bone or poor tissue regeneration capacities [14]. 

Techniques for modifying implant surface 

Titanium implant surfaces can be nano-featured using a variety of ways [15]. 

Physical techniques

Compaction of Nanoparticles

Molecules with a nanoscale can be created by packing titanium dioxide (TiO2) nanoparticles onto titanium surfaces. Without any documented modifications to the material's physical and chemical properties, this approach was claimed to improve osteoblast binding [16].

Grit Blasting

Nanometric designs on titanium implants have been created using a mixture of grit blasting and acid etching processes [17, 18]. Early bone regeneration activities are positively impacted by such surfaces [17, 18]. Blasting materials contain silicon carbide (SiC) or aluminum oxide (Al2O3) [19]. Nanoparticles with various compositions and sizes have the ability to alter the ultimate structure of surfaces [19]. Because of the rapid impact velocities between tiny particles and prosthetic surfaces, nanoparticles can still adhere to the implant even after ultrasonication, passivation using acids, and sterilizing [20, 21].

Chemical modification

By modifying the surface chemicals, it is possible to change the morphology and incorporate chemical components that can improve bioactivity, and anti-corrosion property, and provide surface purification [22]. A conventional acid or soaking can introduce distinct surface chemical properties or topographies, with impressive results [22]. The osteogenic capacity of dental implants has been improved by adding some tens of nanometer range or a couple of micrometers of surface oxide [22].

Hydrogen Peroxide (H2O2)

The creation of a gel-like anatase coating is how H2O2 works to increase the bioactivity of Ti implants [23]. After being submerged in a simulated body fluid (SBF), the anatase allows the bone-like apatite to settle [23]. The modified layer elevates the osteoblast-like structures, which speeds up osteointegration [24]. By combining hydrogen peroxide treatment with acid etching and then chemically heating the CP-Ti sheets up to 800°C, Wang et al. (2002) created an unstructured coating of TiO2 on the material [25]. They came to the conclusion that the novel solution was the ideal solution content and the ideal heating temperature was 400-500°C to achieve the maximum bioactivity [25].

Alkaline Treatment

By forming a sodium titanate gel, dental coatings treated with sodium hydroxide (NaOH) create a nanoscale morphology that reveals the active groups of the layers [26]. This gel can be converted to anatase through straightforward hydrothermal processing, creating, in the researchers' assessment, extremely bioactive areas [27]. NaOH can be applied to materials created using a variety of processes to boost their reaction [28]. As an illustration, nanotubes prepared with NaOH collected through anodization or macroporous Ti surface layer structure through plasma spraying are transformed into a biologically active sodium titanate surface that can result in the creation of apatite, which resembles bone, in an SBF [28, 29]. Materials that have been treated with NaOH can then be silanized to produce antibacterial properties [30]. 

Acid Treatment

By eliminating contaminants and the oxide layer that has formed on the implant, acid cleaning helps to establish a consistent and hygienic surface. Acids including hydrogen Sulfuric Acid (H2SO4), Nitric Acid (HNO3), Hydrofluoric Acid (HF), and Hydrochloric Acid (HCl) are frequently utilized [31]. The chemical treatment produces abrasion, which expands surface area and improves bone-to-implant contact [31]. Takeuchi et al. (2003) assessed the effectiveness of acids - H2SO4, and HCL - decontaminating Ti coatings as preparation for implant surface changes and found that HCl disinfection was a preferable treatment technique [31]. On titanium, CrCoMo (chromium, cobalt, and molybdenum) alloys, Ti6Al4V (titanium, 6% aluminum, and 4% vanadium), tantalum, and nanopit networks, which have pit diameters 20-100 nm, can be successfully created by mixing strong bases or acidic compounds with oxidants [32]. Factors, including etching solution composition, melting temperature, and exposure time, can regulate surface morphology, water sorption, the width of the protective oxide film, and nano roughness [33]. Furthermore, it is feasible to integrate particular materials by changing the nature of the etching solution [33]. When fluorine was added to the implant surface, the growth of both *Porphyromonas gingivalis* and *Aggregatibacter actinomycetemcomitans* was inhibited compared to polished titanium [34, 35].

Modification of Biomolecules

To improve bone-implant interaction and peri-implant bone growth, implants are coated with bioactive substances like peptides or collagen [36, 37]. Chitosan and other naturally biotic and antimicrobial polymers are also employed to alter implant surfaces [38]. 

Mechanical methods

Mechanical techniques were widely applied in the early implant production systems up until the 1990s. Shot peening (SP) and abrasion are applied as surface-improving techniques to progressively smooth the particle size of the outer layer while machining, grinding, refinishing, and sandblasting (SB) are used to alter the layer and improve the overall roughness.

Sandblasting (SB) 

Sandblasting, often referred to as grit blasting, is an approach that is frequently used to manage the texture and change the surface roughness (Ra) levels. Surface morphology produced by SB, regardless of machining, is greatly influenced by the size of particles [39]. SB provides a more advantageous method since it can regulate material texture. This benefit gained attention when Jemat et al. found that the SB technique increased the surface roughness of the implants, which improved their osseointegration ability without compromising the mechanical properties of the implants than on smooth ones [40, 41]. 

Grinding

Different abrasive techniques are used during grinding to enhance the implants' surface. There are typically two techniques that are widely utilized: either using a mechanical arm to operate a belt device or abrading the area using a grinding wheel loaded with abrasive grains. Although the grinding wheel with erosive level 60 can create Ra levels of less than 1 µm, the belt device has a drawback in generating nanoscale structures [42]. In order to enhance the surface's smoothness, Rajeshkumar et al. sandpapered Ti grade-5 utilizing ultrasonic-vibration-assisted minimum quantity lubrication (UMQL) and conventional minimum quantity lubrication (CMQL). According to their findings, contrasted to CMQL, the difference in vegetable oil in UMQL caused differences in density, viscosity, hydrophilicity, and a decline in cutting forces. Yet UMQL's final roughness was a little bit more than CMQL's. This shows that the mechanical properties improved [43]. 

Plasma Spraying

With the help of this technique, a nanoengineered material with proportions under 100 nm can be produced. This procedure starts with the vacuum-assisted removal of surface impurities, then it deposits energized metallic ions or plasma on the top of implants under the direction of kinetic energy. This procedure enables the covering of a variety of materials, including metals, polymers, and ceramics, with different metals including gold (Au), Ti, and silver (Ag). This method is frequently used to apply calcium phosphate coatings (HA) to implant surfaces in order to change their bioactivity. On the nanoscale implant surface, the osteoblast level rises. Implants with hydroxyapatite coatings have a high proportion of bone-implant interaction, as per De Groot et al. (1987) [44].

Anodization

When titanium is subjected to air, a coating of titania (TiO2) is formed. It has a width of 2 to 5 nm and forms on its exterior and prevents corrosion and keeps the substance bioinert [45, 46]. Unfortunately, in rare instances, they lack osseointegration and are encased by fibrous tissue in vivo [47]. Lipopolysaccharides and low pH are additional factors that accelerate the dissolution of titanium [48]. Different surface modifications have been made to promote bioactivity and osteointegration. Recently, the titanium anodization process that produces titanium oxide nanotubes (TNTs) on exterior surfaces has received a lot of interest [49-53]. According to Ganguly et al. (2014), the anodized TNT layer has great possibilities for use in implant dentistry because it has been proven to boost osteoblast cell attachment and desired properties, accelerate the formation of hydroxyapatite, and influence cellular activity to improve tissue integration [49, 50, 53-55]. TNTs coatings have greater hydroxyapatite adherence than non-anodized materials [56]. Cell attachment is enhanced by the adhesion strength of the titanium oxide nanotubular surface with the hydroxyapatite covering. When the oxide layer is thicker, more hydroxyapatite is formed, and crystalline structures form it more frequently than amorphous structures [54]. Numerous research has looked into how mesenchymal stem cells and osteoblast cells react to TNTs.

Laser Ablation

The Laser-Lok®implant (Bophorizons, Alabama, USA) is unique because of its production process, which focuses on enhancing the incorporation of implants with adjacent tissue [57]. As a result, the implant collar production process is applied to nanoscale surfaces [57]. Laser micromachining is used to create a series of micro and nanoscale microchannels on the Laser-Lok implant's neck [57]. These microchannels are thought to function as a biological barrier by encouraging connective cell and bone adhesion and preventing epithelial breakdown [57]. Nevins et al. (2010) used histological samples from a dog model to show that the connective tissue development around Laser-Lok abutments is arranged perpendicularly [57]. It has been suggested that the thick cervical seal functions as a barrier, stopping junctional epithelium from migrating apically [58]. After rapid effective activation, Laser-Lok implants have 2-year survivability of 96.1% (Farronato et al., 2014) [58]. Results must be carefully evaluated, though, due to the lack of any long-term trials [57, 58]. 

Photofunctionalization

By changing the titanium dioxide on the exterior surface, ultraviolet (UV) treatment improves bioactivity and osseointegration [59]. UV light is thought to improve osteoconductivity by encouraging chemical bonding between cells and proteins and the implant surface [59]. UV treatment enhances surface energy and water sorption while decreasing the amount of surface hydrocarbon [60]. It has been demonstrated that bioactivity lost due to aging-related wearing is restored by UV radiation, which has been proposed to increase protein uptake and cell adhesion to titanium coatings [61]. 

Surface modifications and related interactions

Because of their perfectly polished and flat textures, the earlier implants had weaker osseointegration which caused a significant number of implant failures [62]. Presently, studies are centered on developing strategies for surface roughening and bioactivity of zirconia implants [63-65]. Through thermochemical, chemical, and physical surface variation methods, cell activity and functioning have been successfully improved [63]. Nonetheless, zirconia implants generally produce roughness textures that are lower than those of titanium implants [63]. In comparison to zirconia implants, titanium implants continue to have a bit faster rate of bone growth surrounding implants and a stronger bone-implant interaction [64]. Research examining the durable effectiveness of acid and laser-etched ceramic implants is scarce, in comparison to research examining titanium ones [63, 65]. Surface activities and nano interactions both strongly promote the proliferation and adherence of cells [66]. By chemical interactions with the nanoscale layer, outer membrane proteins and molecules can interact more readily, accelerating the attachment, division, and growth of cells [66, 67]. 

Effects of Surface Modification 

The alteration of surfaces at the nanoscale has been shown to affect protein adsorption [68]. Cell selectivity and the degree of cell adherence are also changed [68]. Cell proliferation might well be accelerated or slowed down based on the nanoarchitecture [68]. Numerous researchers have found that nanoscale design improves osteoblast development [68]. It is thought that the early protein-surface interactions regulate the osteoblast attachment phase [65]. The osseointegration phase depends heavily on this factor [68]. Protein adsorption, like plasma fibronectin, which happens as soon as the implants make an interaction with a biological milieu, may facilitate cellular adhesion and differentiation [68]. Integrin receptors, which send signals via focal contacts (a group of proteins on the intracellular surface of the layer), are involved in cell attachment to the protein domains of sticky extracellular matrix proteins [68]. Integrins, which link to the cell-binding proteins, frequently promote epidermal attachments [68]. Cell attachment of osteoblasts and other molecules to layers of synthetic materials is mediated by cell-attaching proteins like fibronectin or vitronectin [66, 68]. These proteins' orientation may be altered by nanofeatures, which are shown to have an impact on cell adherence and behavior [66, 68].

Nanosurface and Surface Reactivity

The surface sensitivity of endosseous implants may be affected by nanosurface changes [69]. In titanium implants, minimal bone adhesion occurs, particularly at the beginning of bone development [69]. In the beginning stages of the healing process, nanoscale morphological alterations frequently influence the chemical interaction of substances and the development of bone on the implanted site [70]. 

Cell Response on Nanosurfaces 

Cells are remarkably adept in detecting nanostructures, regardless of the surface-adsorbed proteins [71]. A surface's nanofeatures have an impact on cell attachment as well as migration [71, 72]. Cell adhesion activity is a factor that influences these cell characteristics [71, 72]. Cell actions are influenced by the morphology of the supporting substratum through indirect and direct contact [73]. There have been distinct interaction spots for the lamellipodia of propagating cells in the 20-40 nm features generated by the H2O2/H2SO4 treatment [71]. Alloplastic surfaces' unique nanostructures may have properties that alter how cells interact [73]. The size and concentration of nanostructures have an impact on cell function [73]. 

Cell Differentiation 

Osteoblast-specific attachment and adhesive cell growth are equally important for the development of osseointegration like the efficient division of adhesive mesenchymal cells in the osteoblast line [74]. Research showed that after 3 and 4 weeks, cell layers grown on nanoparticles produced more alkaline phosphatase and contained more calcium minerals [74, 75]. 

Interactions of Protein and Surface in Nano-surface 

An important step in the osseointegration phases, osteoblast attachment, is thought to be controlled by changes in the first protein bonding [76]. The beginning of protein adsorption (for example, plasma fibronectin) when the implant interacts with a biological milieu encourages further growth and adhesion of cells [76]. Changes in a biomaterial's surface energy or water sorption are standard ways to modify how cells engage with surfaces [77]. A well-established method for changing protein affinities on the material is nano-scale morphology [77]. Comparing normal surfaces to nanostructures, Webster et al. found that vitronectin adsorption was enhanced [77]. 

Table 1 summarizes different ways of implanting nano-featured surface modifications [15]. 

**Table 1:** Techniques for the addition of nanotechnology to dental implants NaOH: sodium hydroxide

Methods	Features
Self-assembly of monolayers	One or more molecules with various functionalities could be the exposed functional end group (a cell adhesive or osteoinductive compound).
Compaction of nanoparticles	preserves the surface's composition across various morphologies. Application over implant materials is difficult.
Ion beam deposition	depending on the material utilized, can imbue the surface with nanofeatures.
Acid etching	Combining this technique with others (such as sandblasting and/or peroxidation) can give the surface nanofeatures and eliminate impurities.
Peroxidation	creates a coating of titanium. Changes in morphology and chemistry are introduced.
Alkaline treatment (NaOH)	creates a sodium titanate gel layer that enables the deposition of hydroxyapatite. Changes are conveyed in terms of both chemistry and topography.
Anodization	can create a new oxide layer by adding nanofeatures to the surface (depending on the material used).
Sol-gel (colloidal particle adsorption)	produces a thin film with carefully controlled chemical properties. Strong physical interactions can be seen in atomic-scale interactions.
Discrete crystalline deposition	creates a surface with a structural complexity at the nanoscale
Lithography and contact printing technique	Various materials and forms can be used to cover the surface. Methods are time-consuming and need to be developed extensively before being applied to implant surfaces and being translated for clinical use.

Recent developments

A developing theory on the etiopathogenesis of peri-implantitis has looked at how nanoparticle discharge as a result of dental implant disintegration can act as an infection trigger [78]. This process has been thoroughly investigated for orthopedic tools, where it was shown that the degradation of metal biomaterials causes exogenous nanoparticles to build up in the peri-implant environment [79]. Similarly, a few researchers claim that destruction to the exterior of devices can be brought on by both exterior equipment and complex interaction at the implant site. Even so, for titanium implants, this can arise from a corrosive environment, however, there is no studies conducted on this aspect [78]. In cases of infection in peri-implantitis and periodontitis, different cell components were observed, with increased macrophage polarization in the first condition [80]. Other histological research, however, found that implantitis concerns are more patient-specific than material-specific and rely on the existence of infections surrounding titanium and ceramic prostheses [81]. 

High-performance liquid chromatography combined with inductively coupled plasma mass spectrometry (HPLC-ICP-MS) was employed to analyze the dispersion and compositional differentiation of metals due to the likelihood of dental implant breakdown within metallic discharge [82]. 

## Conclusions

Numerous methods have been extensively researched and produced to change implant materials in order to enhance osseointegration and tissue regeneration. The chemical and biological reactivity of the dental implant in the mouth is altered via nanosurface modification. This modified behavior has been associated with the different ways in which ions, proteins, and cells connect with the surface of the implant. As a result of these changed connections, molecular and cellular processes that affect osseointegration are positively impacted. Whether this altered behavior can be due to the nanosurface changes alone has to be further investigated. Following their prolonged use in the oral cavity, more analysis and practical testing will reveal the underlying advantages and drawbacks of nanostructure alterations to implant materials. Numerous in vivo and in vitro investigations have shown a variety of innovative materials, the majority of which were revisions of ones that were already available in the market. Nanosurface engineering techniques are anticipated to improve titanium dentistry implants' surface characteristics, which in turn promote peri-implant osteogenesis. Implant surfaces' primary flaw is their production process's subjective aspect, which lacks a unified norm for creating regulated structures. There still need to be many in vitro and in vivo investigations conducted in order to solve this problem. Before realizing its complete capacity, nanotechnology must undergo considerably more research.
